# Head and neck cancer in living donor liver transplant recipients: Single center retrospective study

**DOI:** 10.1097/MD.0000000000016701

**Published:** 2019-08-02

**Authors:** Nan-Chin Lin, Yao-Li Chen, Kuo-Yang Tsai

**Affiliations:** a School of Medicine, China Medical University, Taichung; b School of Medicine, Kaohsiung Medical University, Kaohsiung; c School of Medicine, Taipei Medical University, Taipei, Taiwan, ROC.

**Keywords:** head and neck cancer, living donor liver transplantation, oral cancer

## Abstract

The purpose of this study was to investigate the incidence and risk factors of head and neck cancer in living donor liver transplant (LDLT) recipients.

This is a retrospective cohort study. A case-matched (1:4) comparison between recipients with and without developed head and neck cancer after LDLT was conducted. The differences between 2 groups were analyzed.

The incidence of head and neck malignancy in our cohort was 9 of 453 (1.98%). Their cumulative survival rate was below 60% at 24 months after the diagnosis of head and neck cancer, and no recipients lived for more than 2 years after being diagnosed with stage IV cancer. In the case–control study, univariate analysis revealed that alcohol consumption (odds ratio [OR] = 8.75, 95% confidence interval [CI]: 1.55–49.56) and smoking (OR = 6.71, 95% CI: 1.20– 37.44) were factors associated with the incidence of head and neck cancer after LDLT.

In the conclusion, recipients with head and neck cancer after LDLT may have a rather poor prognosis, especially those who are initially diagnosed with advanced-stage disease. Alcohol consumption and smoking may be the predisposing factors to head and neck cancer in LDLT recipients.

## 1 Introduction

When patients experience liver failure due to chronic liver disease, liver transplantation is a treatment option, although it is not the initial or primary treatment modality for most liver diseases. In 2013, the American Association for the Study of Liver Diseases and the American Society of Transplantation developed guidelines regarding the indications for liver transplantation, including acute liver failure, cirrhosis with complications, certain liver neoplasms, and liver-based metabolic conditions with systemic manifestations.^[1]^

The early challenges to successful liver transplantation included surgical techniques, organ preservation, and immunosuppression. As surgical techniques improved, the need for better immunosuppression became clearer. A report published in 1980 described a 1-year survival rate after liver transplantation of only 26%.^[2]^ The introduction of the calcineurin inhibitor cyclosporine A the following year marked a turning point for liver transplantation.^[3,4]^

In 1994, the United States Multi-Center FK506 Liver Study Group published a report comparing the pros and cons of cyclosporine and tacrolimus for immunosuppression in liver transplantation patients.^[5]^ This study was a keystone in the evolution of liver transplantation protocols. According to this report, rejection is the main contributor to graft loss and death. Furthermore, the study revealed little difference between the survival of transplant recipients receiving cyclosporine and those receiving tacrolimus, but tacrolimus led to fewer episodes of steroid-resistant rejection. However, it was noted that tacrolimus causes many side effects, including nephrotoxicity and neurotoxicity.

Liver transplantation protocols have evolved dramatically since the publication of the 1994 study, and many of the issues outlined in that report have been addressed. Currently, acute rejection is usually easy to manage, though clinicians must balance the risk of rejection with the risk of drug toxicity. The main concern has shifted to preventing the recurrence of liver disease and mitigating the long-term complications of immunosuppression. Tacrolimus has become the 1st-line immunosuppressant for most liver transplantation recipients, and several auxiliary drugs have been adopted for adjusting immunosuppression.

There is an increased risk of a wide range of cancers associated with solid organ transplantation. The most extensive data on this subject come from a cohort study that analyzed the frequency of malignancy in over 175,000 solid organ transplant recipients from 1987 to 2008.^[6]^ The most commonly transplanted organs included the kidney, liver, heart, and lung (in 58%, 22%, 10%, and 4% of cases, respectively). Malignancies were identified in over 10,656 cases, corresponding to a standardized incidence ratio of 2.10 (95% confidence interval [CI]: 2.06–2.14) compared with the general population and an excess absolute risk of 719 cases per 100,000 person-years. Organ transplantation significantly increased the risk of more than 30 distinct primary malignancies, including head and neck cancer. Relative to the general population, organ transplantation led to a 5-fold or greater increase in risk in several tumor sites.

The overall level of immunosuppression has been suggested to be the main factor increasing the risk of posttransplantation malignancy.^[7]^ Data from 1 study suggested that the use of tacrolimus increases the risk of malignancy following solid organ transplantation.^[8]^ Further research showed that tacrolimus has a dose-dependent effect on tumor progression and TGF-beta 1 expression, and this increased expression may be a pathogenetic mechanism underlying tumor progression.^[9]^

The main purpose of this study was to investigate the increased incidence of head and neck cancer postliver transplantation in our institution and to analyze the difference between those recipients who got head and neck cancer and those who did not.

## 2 Methods

### 2.1 Patients

This is a retrospective cohort study. All clinical data were obtained through chart review. A total of 455 patients who received living donor liver transplantation (LDLT) at the Changhua Christian Hospital between January 1, 2005 and December 31, 2017 were enrolled. The study was approved by the institutional review board of the Changhua Christian Hospital. Patients with a history of head and neck cancer before liver transplantation and diagnosed head and neck cancer within 60 days after liver transplantation (n = 2) were excluded. Finally, 453 patients were identified and subsequently analyzed. A case-matched comparison between recipients with and without developed head and neck cancer after LDLT was conducted.

### 2.2 Clinical parameters

Recipient characteristics including age, gender, life behavior, type of immunosuppressive medications used postliver transplantation, the interval from transplantation to cancer diagnosis, cancer therapy, metastasis and local regional recurrence, and incidence of secondary or tertiary cancer development were collected through chart reviews. The diagnosis of head and neck cancer was based on pathology reports. The anatomic site of a head and neck cancer was classified using the International Classification of Disease. Diagnosis was identified by ICD-9 (140–149) and ICD-10 (C00-C14). Among the 453 LDLT recipients, 9 subsequently developed head and neck squamous cell carcinoma (SCC) and received treatment at our hospital. Then we designed an observational, retrospective, case–control study with 1:4 matching.

The 9 recipients who had head and neck cancer after LDLT (group 1) were individually matched to 35 LDLT recipients (group 2) on the basis of gender and age. In all 35 matched recipients, we enrolled those who did not get any malignancies postliver transplantation and were followed for at least 24 months.

### 2.3 Statistical analysis

Continuous variables are presented as mean ± standard deviation and categorical variables are presented as percentages. The Mann–Whitney *U* test was used to compare continuous variables between recipients with head and neck cancer and those without after LDLT. The Chi-squared test was used to compare differences in categorical variables between the 2 groups. Estimates of the overall survival (OS) rates were calculated using Kaplan–Meier analyses. The comparisons of group survival functions were conducted using log rank tests based on the OS. A binary logistic-regression model was used to identify risk factors for the incidence of head and neck cancer after LDLT. A *P*-value of <.05 was considered to represent statistical significance. All statistical analyses were performed with the statistical package SPSS for Windows (Version 16; SPSS Inc, Chicago, IL).

## 3 Results

**Figure 1:**
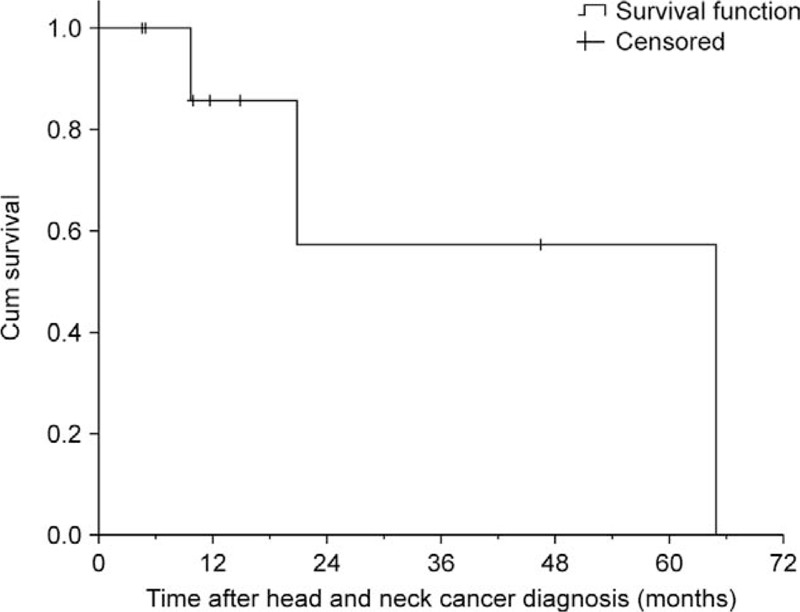
Overall survival of liver transplantation recipients after being diagnosed with head and neck cancer.

Our cohort study enrolled 453 patients, and the incidence of head and neck malignancy after liver transplantation was 9 of 453 (1.98%). In terms of anatomic site, 3 patients developed SCC in the tongue, 1 in the retromolar trigone, 1 in the tonsils, 1 in the larynx, 1 in the buccal mucosa, and 2 in the oropharynx. The mean age at the time of diagnosis of head and neck cancer was 56 years, with an average interval between liver transplantation and cancer diagnosis of 31.3 months. Among the 9 patients diagnosed with head and neck SCC, 8 had advanced stage III or stage IV malignancies (Table 1). Their cumulative survival rate was below 60% at 24 months after diagnosis, and no transplant recipients had survived more than 2 years when head and neck SCC was diagnosed at stage IV (Table 1, Fig. 1). All transplant recipients were treated with tacrolimus after liver transplantation at dosages dependent on blood concentration. In the case–control study, Table 2 shows the results of the analyses of both groups. The proportion of alcohol abuse was almost 3 times higher in group 1 than in group 2 (*P* = .017). The proportion of smoking was 2 times higher in group 1 than in group 2 (*P* = .027). The univariate analysis results in Table 3 reveal that alcohol abuse (odds ratio [OR] = 8.75, 95% CI: 1.55–49.56) and smoking (OR = 6.71, 95% CI: 1.20–37.44) were factors associated with the incidence of head and neck cancer after LDLT. No significant differences were found for the other variables.

**Table 1:**
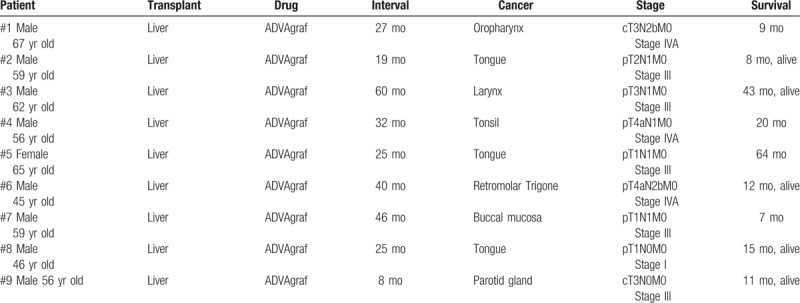
Interval between liver transplantation and cancer diagnosis, as well as the survival time after cancer diagnosis of 9 patients who were diagnosed with head and neck cancer in advanced stages.

**Table 2:**
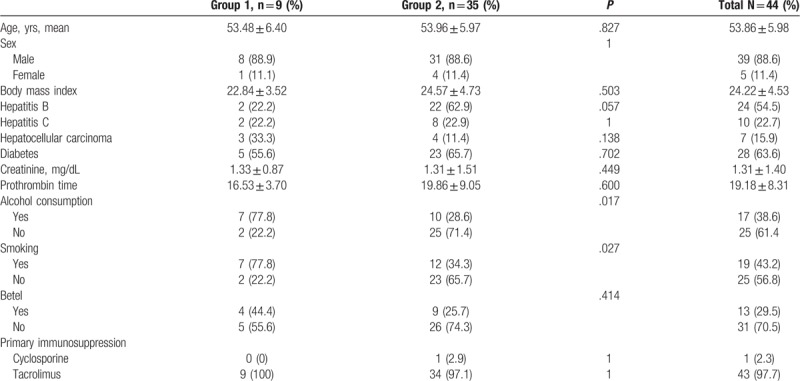
Pretransplant characteristics of the patients in both groups.

**Table 3:**
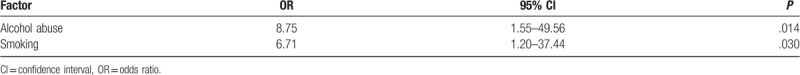
Associated factors of the incidence of head and neck cancer in recipients after living donor liver transplantation.

## 4 Discussion

Several studies have established an increased incidence of de novo cancers after solid organ transplantation.^[10–14]^ As in our study, when de novo cancers emerge they usually become highly aggressive.^[15,16]^ The mean age at which head and neck cancer was diagnosed in our 9 transplant recipients (56 years) was consistent with that of the general population (55–65 years), according to data from the Health Promotion Administration, Ministry of Health and Welfare in Taiwan.^[17]^ The incidence of head and neck cancer in our cohort, 9 of 453 (1.98%), is much higher than that in the general population of Taiwan, which in 2014 was estimated to be 22.9 cases per 100,000 people (0.023%).^[17]^ The increased incidence of head and neck cancer postliver transplantation in our cohort is in agreement with data from previous studies.^[10,16]^ Moreover, the mean interval between liver transplantation and the diagnosis of head and neck cancer in our cohort was 31.3 months, suggesting that these malignancies developed during the period of long-term immunosuppression after solid organ transplantation. Among the 9 patients who developed head and neck cancer, 6 had a significant history of smoking and alcohol consumption. In addition to the immunosuppressed condition of those recipients, it is worth noting that smoking and alcohol consumption also represent significant risk factors for aerodigestive cancer.

In the general population, the 5-year survival rate after the diagnosis of advanced-stage head and neck SCC is 51.8% for stage III and 39.6% for stage IVA, as shown by data from our institution from 2004 to 2014 (Table 4). In the cohort of the present study, the transplant patients who developed head and neck SCC had a much worse prognosis: only one of the 9 was still alive 4 years after head and neck cancer was diagnosed. Similarly, Barrett et al^[15]^ found that transplant recipients diagnosed with cancer in advanced stages have poor prognoses: of 19 patients with advanced disease, only 1 survived and became disease free. Sixteen died of progressive disease at a median of 1 month after diagnosis, and 2 died of intercurrent diseases within 1 week of their diagnoses.

**Table 4:**
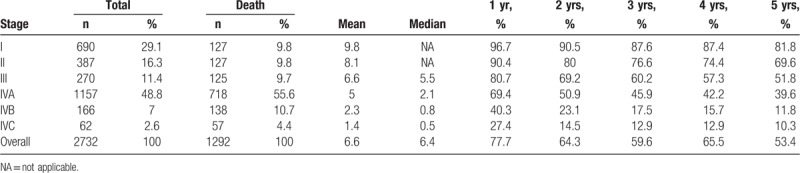
Five-year survival rate from head and neck squamous cell carcinoma in Changhua Christian Hospital between 2004 and 2014.

Regarding tumor recurrence, neck metastasis was pathologically confirmed for 8 of our 9 patients, and 4 of them experienced locoregional recurrence within 1 year. One patient developed a 3rd malignant tumor 2 years after tongue SCC was diagnosed. Thus, solid organ transplantation recipients who are later diagnosed with advanced head and neck squamous cell carcinoma more likely pass away early with standard cancer therapy.

Of the 9 transplant recipients who developed head and neck SCC, 6 developed oral mucosal lesions, 4 had lichenoid lesions, and 1 had erythroplakia before developing oral cancer. The high frequency of oral mucosal lesions among liver transplant recipients may be explained not only by immunosuppressive drugs but also by other medications such as antihypertensives.^[18,19]^ For example, angiotensin-converting enzyme inhibitors, beta-blockers, and diuretics, which are commonly used for liver transplant recipients, can trigger lichenoid drug reactions.^[20]^ As such, doctors should carefully monitor erosive and atrophic oral lichen planus or lichenoid lesions, which have a 0.5% to 4% chance to become malignant.^[21,22]^ A biopsy is the only way to achieve a definite diagnosis of a suspected lesion, for either ulcerative lesions or other lesions that are clinically suspected to have malignant potential.^[23]^ Therefore, annual oral examinations are indicated for liver transplantation recipients, especially for those who develop oral mucosal lesions.^[24]^

There is not yet a definitive best method to care for organ transplant recipients. If immunosuppression is reversed, it may jeopardize the graft. However, once a de novo malignancy has been diagnosed, reversion of immunosuppression will probably have little effect on the outcome. Still, reducing immunosuppression might improve the ability of the immune system to guard against metastasis and recurrence. In the future, protocols for immunosuppressive modification need to be established, balancing the need to prevent malignancies with the need to maintain transplanted organs.

The main limitation of the present study is small sample size that might have prevented us from detecting a statistically significant difference between the study group and the control group. The other drawbacks include its retrospective design, which may increase the risk of case selection. Additionally, we might suffered from some bias regarding the data collected in a single region and some life style include alcohol consumption and smoking may be the same predisposing factors to head and neck cancer both in recipients and general population. Therefore, larger sample size and multicentric studies are warranted to prove our findings.

In our study, a high incidence of head and neck cancer among liver transplant recipients was found; those with head and neck cancer may have a rather poor prognosis, especially those who are initially diagnosed with advanced-stage disease. In conclusion, oral mucosal and upper digestive system examinations may be indicated for those candidates before receiving liver transplantation and become annual examinations. If there is any lesion or premalignant condition found, close follow-up should be carried out.

## Author contributions

**Conceptualization:** Kuo Yang Tsai.

**Data curation:** Nan-Chin Lin.

**Formal analysis:** Nan-Chin Lin.

**Funding acquisition:** Kuo Yang Tsai.

**Investigation:** Nan-Chin Lin.

**Methodology:** Nan-Chin Lin.

**Project administration:** Kuo Yang Tsai.

**Resources:** Nan-Chin Lin, Yao-Li Chen.

**Software:** Nan-Chin Lin.

**Supervision:** Kuo Yang Tsai, Yao-Li Chen.

**Validation:** Kuo Yang Tsai.

**Visualization:** Kuo Yang Tsai.

**Writing – original draft:** Nan-Chin Lin.

**Writing – review & editing:** Kuo Yang Tsai.

## References

[1] Martin P DiMartini A Feng S Evaluation for liver transplantation in adults: 2013 practice guideline by the American Association for the Study of Liver Diseases and the American Society of Transplantation. Hepatology 2014;59:1144–65. 24716201 10.1002/hep.26972

[2] Starzl TE Koep L Porter KA Decline in survival after liver transplantation. Arch Surg 1980;115:815–9. 6992740 10.1001/archsurg.1980.01380070009002 PMC2962607

[3] Starzl TE Klintmalm GB Porter KA Liver transplantation with use of cyclosporin a and prednisone. N Engl J Med 1981;305:266–9. 7017414 10.1056/NEJM198107303050507 PMC2772056

[4] Calne RY Liver transplantation. Ann Clin Res 1981;13:327–35. 7039485

[5] U.S. Multicenter FK506 Liver Study Group. A comparison of tacrolimus (FK 506) and cyclosporine for immunosuppression in liver transplantation. N Engl J Med 1994;331:1110–5. 7523946 10.1056/NEJM199410273311702

[6] Engels EA Pfeiffer RM Fraumeni JF Jr Spectrum of cancer risk among US solid organ transplant recipients. JAMA 2011;306:1891–901. 22045767 10.1001/jama.2011.1592 PMC3310893

[7] Buell JF Gross TG Woodle ES Malignancy after transplantation. Transplantation 2005;80:S254–64. 16251858 10.1097/01.tp.0000186382.81130.ba

[8] Imao T Ichimaru N Takahara S Risk factors for malignancy in Japanese renal transplant recipients. Cancer 2007;109:2109–15. 17407138 10.1002/cncr.22636

[9] Maluccio M Sharma V Lagman M Tacrolimus enhances transforming growth factorbeta1 expression and promotes tumor progression. Transplantation 2003;76:597–602. 12923450 10.1097/01.TP.0000081399.75231.3B

[10] Collett D Mumford L Banner NR Comparison of the incidence of malignancy in recipients of different types of organ: a UK Registry audit. Am J Transplant 2010;10:1889–96. 20659094 10.1111/j.1600-6143.2010.03181.x

[11] Penn I Cancers complicating organ transplantation. N Engl J Med 1990;323:1767–9. 2247108 10.1056/NEJM199012203232510

[12] Penn I Tumors of the immunocompromised patient. Annu Rev Med 1988;39:63–73. 3285791 10.1146/annurev.me.39.020188.000431

[13] Penn I Post-transplant malignancy: the role of immunosuppression. Drug Saf 2000;23:101–13. 10945373 10.2165/00002018-200023020-00002

[14] Sloan GM Cole P Wilson RE Risk indicators of de novo malignancy in renal transplant recipients. Transplant Proc 1977;9:1129–32. 325739

[15] Barrett WL First MR Aron BS Clinical course of malignancies in renal transplant recipients. Cancer 1993;72:2186–9. 8374876 10.1002/1097-0142(19931001)72:7<2186::aid-cncr2820720720>3.0.co;2-2

[16] Penn I Occurrence of cancers in immunosuppressed organ transplant recipients. Clin Transpl 1998;147–58. 10503093

[17] Health Promotion Administration, Ministry of Health and Welfare. Cancer Registry Annual Report, 2015 Available at: https://www.hpa.gov.tw/Pages/List.aspx?nodeid=119 [Accessed 27 December 2018].

[18] Petti S Polimeni A Berloco PB Orofacial diseases in solid organ and hematopoietic stem cell transplant recipients. Oral Dis 2013;19:18–36. 22458357 10.1111/j.1601-0825.2012.01925.x

[19] Scully C Beyli M Ferreiro MC Update on oral lichen planus: etiopathogenesis and management. Crit Rev Oral Biol Med 1998;9:86–122. 9488249 10.1177/10454411980090010501

[20] Ismail SB Kumar SK Zain RB Oral lichen planus and lichenoid reactions: etiopathogenesis, diagnosis, management and malignant transformation. J Oral Sci 2007;49:89–106. 17634721 10.2334/josnusd.49.89

[21] Warnakulasuriya S Johnson NW van der Waal I Nomenclature and classification of potentially malignant disorders of the oral mucosa. J Oral Pathol Med 2007;36:575–80. 17944749 10.1111/j.1600-0714.2007.00582.x

[22] Montero PH Patel PD Palmer FL Changing trends in smoking and alcohol consumption in patients with oral cancer treated at Memorial Sloan-Kettering Cancer Center from 1985 to 2009. Arch Otolaryngol Head Neck Surg 2012;138:817–22. 22986714 10.1001/archoto.2012.1792

[23] Mattsson U Jontell M Holmstrup P Oral lichen planus and malignant transformation: is a recall of patients justified? Crit Rev Oral Biol Med 2002;13:390–6. 12393758 10.1177/154411130201300503

[24] Helenius-Hietala J Ruokonen H Grönroos L Oral mucosal health in liver transplant recipients and controls. Liver Transpl 2014;20:72–80. 24142471 10.1002/lt.23778

